# Interleukin-22 and connective tissue diseases: emerging role in pathogenesis and therapy

**DOI:** 10.1186/s13578-020-00504-1

**Published:** 2021-01-06

**Authors:** Xiuyun Xuan, Lin Zhang, Chunxia Tian, Ting Wu, Haihua Ye, Juanmei Cao, Fangqi Chen, Yan Liang, Huilan Yang, Changzheng Huang

**Affiliations:** 1 Department of Dermatology, Union Hospital, Tongji Medical College, Huazhong University of Science and Technology, Wuhan, 430022 Hubei China; 2 Department of Gerontology, Jinan City People’s Hospital, Jinan, 271199 Shandong China; 3 Department of Integrated Traditional Chinese and Western Medicine, Union Hospital, Tongji Medical College, Huazhong University of Science and Technology, Wuhan, 430022 China; 4 Department of Dermatology, General Hospital of Southern Theatre Command, Guangzhou, 510000 China

**Keywords:** Connective tissue diseases, Interleukin-22, Cytokine, IL-22R, Signalling pathway

## Abstract

Interleukin-22 (IL-22), a member of the IL-10 family of cytokines, is produced by a number of immune cells involved in the immune microenvironment of the body. IL-22 plays its pivotal roles by binding to the IL-22 receptor complex (IL-22R) and subsequently activating the IL-22R downstream signalling pathway. It has recently been reported that IL-22 also contributes to the pathogenesis of many connective tissue diseases (CTDs). In this review, we will discuss the role of IL-22 in several CTDs, such as system lupus erythematosus, rheumatoid arthritis, Sjögren’s syndrome, systemic sclerosis and dermatomyositis, suggesting that IL-22 may be a potential therapeutic target in CTDs.

## Introduction

IL-22, one number of the IL-10 family, is known for its dual character: protection and pathogenesis. IL-22 is part of the defence against pathogens and contributes to wound healing and regeneration. Meanwhile, like other IL-10 family numbers, IL-22 participates in pathogenic processes [1], including in various common malignancies (colon cancer, liver cancer, gastric cancer, breast cancer, pancreatic cancer and cutaneous cancer) [2]. The roles mediated by IL-22 in the inflammatory microenvironment are mutable; thus, it can yield either protective or pathogenic functions [3].

Recently, increasing amounts of evidence regarding the impacts of IL-22 in CTDs are emerging. CTDs are a group of diseases that involve multiple systems and organs, seriously affecting the quality of life of affected patients. However, there are no drugs that can cure CTDs. IL-22 and IL-22-producing cells have been reported to be involved in CTDs [4–6]. In this review, we gathered and discussed current studies on the impacts and latent mechanisms of IL-22 in CTDs (i.e., SLE, RA, SS, SSc and DM), aiming to provide new therapeutic targets for CTDs.

## The cellular sources of IL-22

IL-22 is produced by some sub-T cells, including Th1, Th17 and Th22 cells, but it is primarily secreted by Th1 cells [7]. With the discovery of other Th cells, Th17 and Th22 cells are now thought to be main IL22-producers, and the IL-22-secreting cells tend to gather on barrier surfaces [8]. Th17 cells secret both IL-17 and IL-22, but their expression patterns differ. Transforming growth factor-β (TGF-β) is an inducer of IL-17 and an inhibitor of IL-22 [9]. Moreover, the transcription factors that regulate their expression are different. IL-17 is dependent on the nuclear hormone receptor transcription factors retinoic acid-related orphan receptor γt (RORγt) and RORα, while the expression of IL-22 needs the presence of the ligand-dependent transcription factor aryl hydrocarbon receptor (AHR) [10, 11]. IL-17 is the effector cytokine of Th17 cells, while IL-22 is the main mediator of Th22 cells. Th22 cells have been demonstrated to express IL-22, without IL-17 and TNF-a [8], playing an important role in inflammation and in maintaining skin homeostasis.

In addition, CD8^+^ T cells have been demonstrated to secrete IL-22 [7]. Elevated secretion of IL-22 by CD8^+^ T cells has been detected in the inflamed skin of patients with atopic dermatitis, with a positive correlation with disease severity [12]. Notably, γδ T-cells are activated in pulmonary immune responses, expressing IL-22 [13].

Interestingly, innate immune cells are able to secret IL-22. NK-22 cells located in mucosa-associated lymphoid tissue in humans have been shown to have the capacity to produce IL-22 in response to IL-23 [10]. NK-22 cells may act as protectors of mucosa-associated lymphoid tissue via the expression of IL-22 [14].

Furthermore, innate lymphoid cells (ILCs) have been described to have the capacity of secreting IL-22. These IL-22-expressing ILCs reside at barrier surfaces, where they can produce IL-22 in response to IL-23 [3]. Recently, these populations were verified to play a vital role in facilitating innate immunity and intestinal inflammation and to be an original modality of IL22-secreting cells involved in adaptive immunity [15].

## IL-22 acts via the IL-22R pathway

### IL-22 receptor complex

IL-22 has been noted to act in several diseases by binding to heterodimeric receptor complexes, including IL-10R2 and IL-22R1. The former, as one of cytokine IL-10 receptors, is ubiquitously expressed. However, the latter, the specific receptor of IL-22, is restricted to the surface of cell lineages of a non-hematopoietic origin, such as the pancreas, liver, kidney, and barrier surfaces (i.e., skin, lung and intestine) [8]. In addition, Jones et al., found that IL-22 has a high affinity for IL-22R1 [1], while it has a very low capacity for binding to IL-10R2. Interestingly, IL-10R2 is able to bind to the IL-22/IL-22R1 complex with a moderate affinity, after which IL-22R downstream signalling is activated. Interestingly, the expression of IL-22R1 can be adjusted by INF-γ and TNF-α in human skin cells, or concanavalin A or lipopolysaccharide (LPS) stimulation of hepatocytes [16], indicating the action of IL-22 may involve dynamic changes that are related to IL-22R1 expression.

In addition to the IL-22 receptor complex, a soluble IL-22 binding protein (IL-22BP) has been discovered. A homology of 33% was detected between IL-22BP and IL-22R1, but IL-22BP showed higher affinity to IL-22 than IL-22R1. In other words, IL-22BP has a capacity of neutralizing nearly all IL-22 activity via binding to IL-22. Similarly, for IL-22R1, the expression of IL-22BP in tissues can also be regulated. For example, elevated IL-22 levels were detected in murine models during the active period of infection, while a decreased IL-22BP level was observed [17]. This evidence indicates that IL-22BP may regulate IL-22 activity *in vivo*. However, additional studies are needed to clarify the underlying mechanism of how IL-22BP regulates IL-22 activity, which can provide new insights into IL-22 as a therapeutic target.

### IL-22R downstream signal transduction pathways

IL-22 binds to IL-22R1, then IL-10R2 binds to the IL22-IL22R1 complex, leading to the activation of a series of downstream signal transduction pathways. Primary investigations showed that Signal Transducer and Activator of Transcription (STAT) 3 was phosphorylated by IL-22R in a murine kidney cell line, while STAT5 was phosphorylated to a lesser extent. However, other studies indicated that the phosphorylation of STAT1, STAT3, and STAT5 occurred in a human kidney cell line [18]. A later study suggested that Janus kinase-1 (JAK1) and tyrosine kinase receptor-2 (TYK2) were involved in IL-22 signalling, triggering the downstream signal transduction pathways, such as the MAPK pathways (i.e., ERK1/2, MEK1/2, JNK, and p38 kinase) and STAT1, STAT3, and STAT5 [19]. The IL-22 receptor complex (IL-22R1 and IL-10R2), belonging to the class II cytokine receptor family, generally activates the JAK-STAT signalling pathway. In addition, STAT3 is primarily phosphorylated in response to IL-22 activity.

**Fig. 1:**
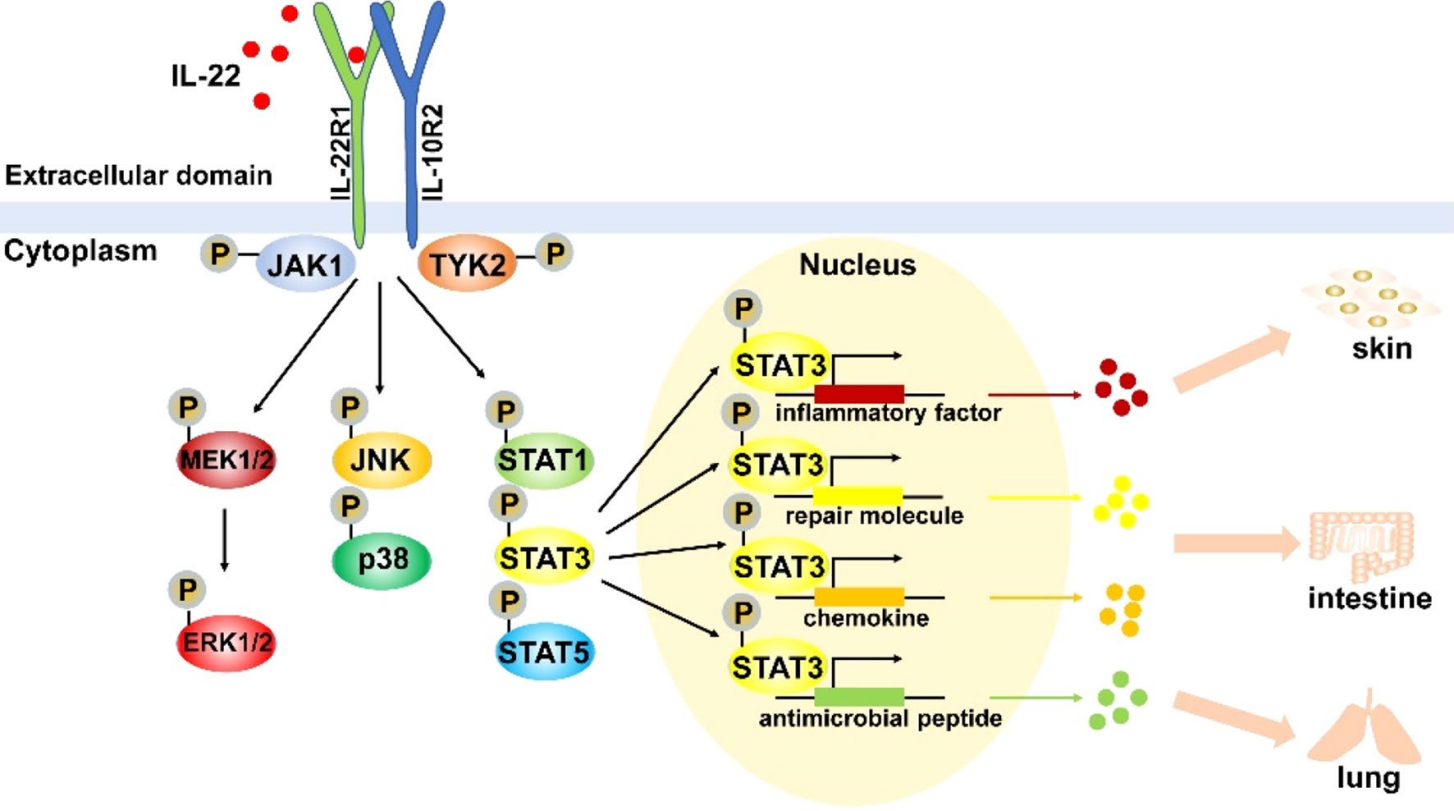
IL-22R downstream signal transduction pathways. IL-22 binds to IL-22R1, then IL-10R2 binds to the IL22-IL22R1 complex, which activates a cascade of downstream signal transduction pathways. IL-22 stimulation activates JAK1 and TYK2, such as the MAPK pathways (i.e., ERK1/2, MEK1/2, JNK, and p38 kinase depending on the cell type), STAT1, STAT3, and STAT5. Among them, STAT3 is the main activated pathway. The above signalling pathways are phosphorylated depending on the IL-22 stimulation. Several mediators are produced after STAT3 phosphorylation, including inflammatory factors, tissue repair molecules, chemokines and antimicrobial peptides, which are involved in many diverse processes in innate immunity and adaptive immunity at barrier surfaces (i.e., skin, intestine and lung). Thus, IL-22 can regulate the process of inflammation, tissue repair, chemotaxis and antimicrobial peptides production depending on the mediators generated and the tissues where it is expressed

However, IL-22R1 signalling shows several unique properties. For example, tyrosine residues on STAT3 are phosphorylated in response to IL-10, while both tyrosine and serine residues on STAT3 are enabled, and then the ERK1/2 pathway is strongly activated in response to IL-22 stimulation [19]. These differences may stem from differences between IL-10R1 and IL-22R1. Moreover, STAT3 signalling is the main signalling pathway involved in IL-22 activity on epithelial cells at barrier surfaces [20]. Similarly, IL-22R signalling, especially STAT3, plays a pivotal role as a prompter of antimicrobial immunity, inflammation and tissue repair at barrier surfaces (i.e., the skin, intestine and lung) in a study of mouse model systems, with the production of molecules associated with inflammation, repair, chemotaxis, and antimicrobial peptides [21] (Fig. 1). Given this, we speculate that IL-22 and IL-22R signalling may affect several related molecules involved in CTDs; thus they are potential therapeutic targets for CTDs.

## IL-22 in CTDs

Just as its name implies, connective tissue diseases (CTDs) mainly damage connective tissues, which involve multitudinous organs and tissues. Patients with CTDs have a low quality of life, and yet, there have not been any curative drugs developed to treat CTDs. The aetiology of CTDs is not clear. They may be the result of an interaction among genetic, environmental and other factors. It has been reported that some immune cells and related cytokines may contribute to CTDs [4, 22]. Among these, IL-22 is a crucial mediator involved in the pathogenesis of CTDs. Previous data indicated that the IL-22 level was elevated in serum samples from RA patients [23], while downregulated plasma IL-22 levels were found in SLE patients [24]. These data showed that multiply pathogenic mechanisms involving IL-22 may contribute to CTDs, depending on the diverse mediators produced and the tissue microenvironment. In this review, we will discuss the pathogenesis of IL-22 and potential therapy targets in connective tissue diseases such as systemic lupus erythematosus (SLE), rheumatoid arthritis (RA), Sjogren’s syndrome (SS), dermatomyositis, and systematic sclerosis (SSc).

### SLE

Systemic lupus erythematosus (SLE) is a common type of CTD, mainly occurring in the population of women of childbearing age. It is characterized by the presence of multiply autoantibodies against several autoantigens, resulting in tissue damage to multi-systems and organs, including the kidneys, joints, skin, and central nervous system [25]. In addition to a mass of autoantibodies produced by unduly activated and proliferative B lymphocytes, sub-populations of T lymphocytes and related cytokines are also involved in the pathogenesis of SLE [26]. Cytokine IL-22 has been reported to play a crucial role in SLE pathogenesis [6].

#### The pathogenesis of IL-22 in SLE

Interestingly, IL-22 expression in SLE is disparate. It has been shown that the IL-22 level is elevated in peripheral blood samples from SLE patients with sole lupus skin (LS) disease while a decreased IL-22 level was observed in lupus nephritis (LN) patients [27]. Similarly, the serum level of Th22 cells was detected to have a similar variation tendency to that of IL-22 [27]. Thus, we speculate that Th22 cells might regulate skin inflammation via excitation of the IL-22/STAT3 pathway in LS disease, secreting multiple mediators including a variety of chemokines and antimicrobial peptides [19, 27]. In the skin, the expression of CCR4 and CCR10, which bind to skin-homing receptors on the surface of Th22 cells, might imply that Th22 cells will inevitably migrate to the skin, leading to increased IL-22 levels in the skin. In addition, cytokine TNF-α, produced by Th22 cells, plays a crucial role in LS disease. It is able to boost the response of keratinocytes to IL-22 by up-regulating the expression of IL-22R1 on the keratinocytes and triggering its downstream signal transduction pathways [28], further amplifying and/or extending these IL-22 effects. Moreover, TNF-α can also activate intercellular adhesion molecule 1 (ICAM-1) expression, prompting dendritic cells (DCs) migration and lymphocyte infiltration [29], any of which can contribute to skin lesions.

On the contrary, in the kidney of LN patients, the expression of Th22 cells and IL-22, to a large extent, were found to be reduced. The differentiation of Th22 cells was caused by DCs in a dependent IL-6 and TNF-α manner. However, a decreased TNF-α level was observed in LN [30], which minimized the development and the effects of Th22 cells. Meanwhile, Cathy et al. found that the expression of urinary IL-22 mRNA is downregulated in patients with proliferative LN [22]. These data indicated that IL-22 might act as a protector in LN. However, in a later study, IL-22 expression was increased in both blood and kidney tissue samples from LN patients and MRL/lpr mice, demonstrating that the increased serum IL-22 levels cannot be due to the IL-22 status in LN, because serum expression of IL-22 would make a difference depending on the different pathogenic forms of LN [31]. To evaluate the role of IL-22 in LN, MRL/lpr mice (a mouse model of lupus), prior to the onset of LN, were treated with anti-IL-22 mAb. The experimental results indicated that anti-IL-22 mAb treatment contributed to less proteinuria, better renal function and less cellular infiltration in the kidney tissues of LN [31]. These results suggest that IL-22 might additionally be a pathogenic cytokine in LN. Nevertheless, further studies are needed to expound on the precise mechanisms of IL-22 in SLE.

In addition, SLE may involve the disruption of self-tolerance, in which TGF-β1 plays a critical role [32]. Raja et al. suggested that an excessive excitation of the IL-22/AhR pathway with the stimulation of multiply environmental stress factors may induce T cells that are resistant to the immunomodulatory effects of TGF-β1, bringing about a loss of the tolerance balance in the active period of SLE [32]. A diminished response to TGF-β1 is involved in the disease activity of SLE, potentially through destroying the PD-1 pathway, which can lead to SLE. More recently, it has been reported that genetic variations of IL-22 can give rise to SLE [33], indicating that IL-22 might help identify new therapeutic targets for SLE. Moreover, copy number variations (CNV) of the IL-22 gene may help screen out the populations who are highly susceptible to SLE [34] (Table 1).

**Table 1:** IL-22 dysregulated in CTDs and possible underlying mechanisms

CTDs	Samples examined	Changes of IL-22 level	Research model	Mechanism	Refs.
SLE	PBMCs	↑	Human (LS)	Up-regulate the expression of IL-22R1 on the keratinocytes	[28]
				Activate ICAM-1 by TNF-α	[29]
		↓	Human (LN)	Decrease TNF-α level	[30]
		ND	Human	CNV of IL-22 gene	[34]
	Serum/kidney	↑	Human (LN), MRL/lpr mice	ND	[31]
	T cells	↑	Human	Destroy PD-1 pathway by TGF-β1	[32]
RA	Synovial tissues	↑	Human, C57BL/6 mice	Enhance the production of MMP1 and S100A8/A9	[42]
				Stimulate production of MCP-1	[48]
				Increase pro-inflammatory mediators	[49]
				Suppress anti-inflammatory IFN-ɣ responses	[50]
				Heighten IL-22 effects by TNF-ɑ, exogenous and endogenous ligands of TLR3 and TLR4	[42]
SS	Salivary glands	↑	Human	Product several cytokines and chemokines (i.e. CXCL13 and CXCL12)	[65]
				Aberrant expression of IL-22R1 on haematopoietic cells	[66]
SSc	Skin	↑	Human	The TNF/IL-22 axis and their receptors, together with TGF-β, in part, constituted the existing inflammatory component of SSc	[72]
				Downregulate let-7a	[77]
DM	Muscle biopsies	↑	Human	IL-22/IL-22R/STAT3 pathway	[88]

↑: upregulation; ↓: downregulation; *ND* not determined

However, there are inconsistencies in the results of several studies of IL-22 expression in SLE patients. Lin J et al. found that IL-22 was closely related to disease activity and ESR of SLE patients in the new-onset phase [24], while Pan H et al. thought that there was no association between IL-22 and the disease activity of SLE [35]. This difference may be due to studying populations of patients in different phases of the disease. Indeed, Lin J et al. concentrated on new-onset patients with SLE, while Pan H et al. recruited only patients who were receiving glucocorticoids treatment. Moreover, plasma IL-22 and serum CRP levels in SLE patients were markedly higher than that in the controls in Zhao L et al.’s study [36], which are contradictory to previous studies showing that plasma IL-22 and CRP levels are lower in SLE patients than that in healthy controls. The difference between Zhao L et al.’s study and the others might be relevant to the different populations of patients recruited, the disease severity and duration, as well as the therapeutic status. Alternatively, the levels of plasma IL-22 and CRP may change during the different periods of SLE pathogenesis. Moreover, Ziesche´ et al., [37] suggested that IL-22 production can be suppressed by the glucocorticoid dexamethasone (DEX) in the context of acute bacterial infections of SLE patients, while McKinley et al. [38] showed that IL-22 production was not responsive to DEX treatment at different doses. Ziesche´ et al. surmised that these diverse results may reflect disparate molecular mechanisms that IL-22 mediated in SLE patients. There are some possible interpretations of these phenomena. The patients recruited were mostly those whose duration of diagnosis was less than 2 months, and these patients probably did not receive long-term glucocorticoid treatment. Furthermore, patients who had a higher SLE disease activity index score may have received higher doses of glucocorticoid treatment. Additional studies are needed to verify these findings, to explore the potential mechanisms and the therapeutic potential of IL-22 in SLE.

#### Regulation of abnormal expression of IL-22 in SLE

Abnormal regulation of IL-22 is contributing to SLE, indicating that targeting IL-22 can become a potential remedy for SLE. As we all know, the main treatment for SLE is immunoregulation. Glucocorticoids (GC) and immuno-depressant agents are the standard treatments for SLE. Zhao et al. found that IL-22 expression was decreased after treatment with GC, cyclophosphamide (CYC) and hydroxychloroquine (HCQ) [39]. Their study showed that the frequencies of the Th22 and Th17 cells, and the levels of IL-22 and IL-17, were decreased after treatment with GC, CYC and HCQ for 4 weeks in a large population of SLE patients. No significant differences in the concentrations of IL-22 and IL-22^+^CD4^+^ T cells were observed in both healthy controls and non-responders either before or after therapy. GC, CYC and HCQ treatment can alleviated the abnormal expression of IL-22, possibly by correcting the polarizations of the IL-22^+^CD4^+^T cells in SLE pathogenesis. These results imply that targeting IL-22 dysregulation may provide a new therapeutic approach to SLE.

### RA

Rheumatoid arthritis (RA) is one type of chronic, inflammatory CTD, characterized by the destruction of polyarticular joints, leading to functional disability, premature mortality, a long-term financial burden and a low life quality [40]. Meanwhile, RA involves diverse pathogenetic factors, including genetic factors, environmental factors and immunological factors. Recently, it has been reported that a complex regulatory network, including a variety of proinflammatory cytokines and chemokines, may be directly implicated in the specific immunological processes of RA, boosting chronic inflammation and joint destruction [41]. Notably, studies have shown that cytokine IL-22 is of great importance in RA pathogenesis [42].

#### The pathogenesis of IL-22 in RA

Fibroblastlike synoviocytes (FLSs) act as a bridge between the inflammatory and destructive phases in RA pathogenesis, through generating numerous chemokines, cytokines, and matrix-degrading enzymes with the stimulation of multiple pathways [43]. In addition, increased IL-22 and IL-22R1 expression were observed in the synovial tissues, and either the lining or sublining layers of the rheumatoid synovium from patients with RA, respectively [44]. Thus, we speculate that IL-22 may be caused the proliferation of FLSs and the production of regulators in RA pathogenesis via multiple mechanisms, including Toll-like receptor (TLR) agonists and proinflammatory mediators [45]. Both of them, resident in the impaired joint, upregulate the expression of the IL-22/IL-22R1 axis present in FLSs and enhance the production of matrix metalloproteinases I (MMP1) and S100A8/A9, aggravating inflammation and destruction of the joint [42]. Among them, alarmins S100A8/A9 have been considered to regulate the cytoskeleton, cell migration and inflammation [46]. In RA mice models, S100A8/A9 were verified to have a pivotal role in the regulation of cartilage destruction and joint inflammation [47].

Meanwhile, signalling pathways involved in the transcription of MMP1 and S100A8/A9 alarmins were examined. Their results suggested that the STAT3, ERK1/2, and p38 MAPK pathways were involved in the production of IL-22-induced S100A8/A9 in FLSs, and were activated to stimulate the proliferation of FLSs and the production of monocyte chemoattractant protein 1 (MCP-1), further intensifying the process of inflammation in RA patients [48]. In addition, pro-inflammatory mediators, including IL-17, IL-1β, TNF-ɑ, IL-6 and MMP-9, were increased in response to IL-22, while the anti-inflammatory IFN-ɣ responses were suppressed [49, 50]. Moreover, TNF-ɑ along with exogenous (i.e., bacterial products, cytomegalovirus) and endogenous ligands (i.e., nucleic acids in necrotic debris, hyaluronan and fibronectin fragments) of TLR3 and TLR4 were able to heighten the IL-22 effects in the joint microenvironment of RA patients [42] (Table 1).

Furthermore, IL-22 expression was highly correlated with rheumatoid factor (RF). Higher levels of IL-22 were observed in patients with bone erosions, demonstrating that IL-22 has the potential to act as a predictive marker of bone destruction in RA [51].

#### Regulation of abnormal expression of IL-22 in RA

Abnormal expression of IL-22 contributes to CTDs as mentioned above, thus IL-22 has potential as a therapeutic target for these disorders. The expression of the IL-22/IL-22R1 axis was detected to be increased in RA FLSs in response to stimulation with TNF-ɑ [42]. Similarly, IL-22/IL-22R1 generation were markedly increased by TLR3 and LPS treatment [42]. The levels of IL-22 and IL-22R1 were elevated by TNF-ɑ, TLR3, and LPS treatment in FLSs. However, their expression was inhibited in the presence of vasoactive intestinal peptide (VIP) [42]. In addition, the IL-22 level can be weakened by simvastatin treatment [52]. PPARɣ, as a nuclear receptor activator, was able to suppress pro-inflammatory gene expression [53]. Similarly, TM17, a thiazolidinedione analogue, has anti-inflammation activity [53]. Notably, IL-22 and IL-17 production by memory (CD4^+^CD45RO^+^) T cells were inhibited when Th17 polarization was modulated by 1,25(OH)_2_D_3_ in RA patients [54]. Moreover, rituximab was able to downregulate IL-22 expression in the impaired joints of RA patients [55]. In their study, a clear Th17 response was induced by *C. albicans*, a potent Th17-inducing stimulus, in the PBMCs of RA patients, while IL-22 production was reduced 20–30% after rituximab treatment. A recent study showed that norepinephrine (NE) could downregulate IL-22 expression [56]. Their study showed that NE reduced the CIA-induced CD4^+^T cell shift towards the Th17 phenotype, further decreasing IL-22 production. Furthermore, higher serum levels of IL-22 were discovered in collagen-induced arthritis (CIA) mice injected with anti-CD3 antibody compared with that in wild-type mice [57]. IL-22 was secreted by lymphocytes freshly isolated from CIA mice in response to anti-CD3 or IL-23 [57, 58]. Similarly, *M. tuberculosis* stimulation also led to IL-22 production [57, 58]. A later study suggested that cigarette smoke consistently led to increased production of IL-22 in RA patients through downregulating the activity of the serine-threonine kinase ROCK2 and the phosphorylation of Interferon Regulatory Factor 4 (IRF4), a known negative regulator of IL-22 production [59]. Interestingly, IL-23 and IL-1 treatment lead to a markedly increased IL-22 level, whereas IL-1Ra, as the natural receptor antagonist of IL-1, reduces IL-22 generation that is IL-1-enhanced [58]. Taken together, these findings indicate that IL-22 regulation may provide new insight into RA therapy.

### SS

Sjögren’s syndrome (SS) is a chronic inflammatory CTD involving the exocrine glands, mainly the lacrimal gland and salivary glands. SS is divided into two types by its aetiology: primary SS (SSp) and secondary SS. The former occurs in isolation, and the latter generally occurs in association with other CTDs, mainly RA [60]. The aetiology of SS is not clear yet. It has been reported that higher levels of some cytokines and chemokines have been observed in blood samples and the inflamed salivary glands of SS patients [61]. The innate and adaptive immune systems play a crucial role in SS pathogenesis. It has been demonstrated that B lymphocytes could produce autoantibodies and several cytokines, and they constitute the important parts of germinal centres in the inflamed salivary glands [62]. In addition to the activation of B cells, several subunits of T lymphocytes, mainly Th1, Th22 and Th17, are activated in the local immune response in pSS patients [63]. Notably, these subunits of T cells secrete cytokine IL-22, which is known to act as a pivotal mediator in T cell-mediated inflammatory disorders [64]. Thus, we reviewed current investigations about the pathogenesis of IL-22 in SS, and provided new insight into therapeutic targets for SS.

#### The pathogenesis of IL-22 in SS

It was demonstrated that IL-22 is overexpressed at either the mRNA or protein level in the inflamed salivary glands samples from pSS patients [65]. IL-22 may act as a prompter in tissue and systemic inflammation. In the same study, we also found that IL-22 together with IL-17 synergistically seemed to exacerbate the process of SS, and that IL-22 played a leading role in the pyogenesis of proinflammation [65]. In SS, IL-22 from the surface barrier may activate an inflammatory response with unknown external stimuli, recruiting inflammatory cells attracted by the production of several cytokines and chemokines (i.e., CXCL13 and CXCL12) [65]. It is well-known that IL-22 acts via binding to the IL-22 receptor, and IL-22R1 expression is restricted to non-haematopoietic cells under physiological conditions [19]. However, F. Ciccia et al. showed that, in pSS patients, IL-22R1 expression aberrantly occurs on haematopoietic cells at either the local or systemic levels [66]. Interestingly, strong phosphorylation of STAT-3 was observed in these haematopoietic cells. In other words, an autocrine IL-22 stimulatory loop exists in pSS [66]. In SS, there is dysregulation of IL-22 and aberrant expression of IL-22R1 on the surface of B and T cells [67], implying that they might act as a stimulatory pathway that can affect the self-perpetuation of the disease and perhaps explain why some SS patients progress to non-Hodgkin’s lymphoma (Table 1).

In addition, we also found that higher expression of IL-22 in both the sera and saliva samples from SS patients was positively related to elevated antibodies, RF and hypergammaglobulinemia, indicating that IL-22 has the potential to act as a biological marker of disease activity [65].

#### Regulation of abnormal expression of IL-22 in SS

Abnormal expression of IL-22 contributes to SS, and thus IL-22 potentially acts as a therapeutic target for this disorder. Kyeong et al. reported that anti-high mobility group box 1 (anti-HMGB1), in NOD.B10.H2b mice, was able to attenuate the clinical symptoms of SS [68]. HMGB1, a cytokine released from necrotic cells or macrophages and DCs, could mediate the response to infection, injury, and inflammation [69]. In their study, corneal epithelial erosions were attenuated, and tear secretion was increased, after treatment with 2 µg or 0.02 µg anti-HMGB1 (p < 0.05). Their results involved the upregulation of ILC3 cells and IL-22, without influencing Th17 cells or Tc17 cells. This is because ILC3 cells play a crucial role in epithelial homeostasis, wound healing and immune modulation [70]. However, it is well-established that ILC3 cells are able to protect epithelial homeostasis by secreting IL-22 in the gut, but their role in SS has not been investigated yet [70]. On the contrary, Francesco et al. found that the IL-22 level and the number of IL-22^+^ cells infiltrated in the inflamed salivary glands of SS patients were significantly reduced after rituximab treatment [71]. Moreover, the function of the two main exocrine glands involved in SS, the salivary gland and the lacrimal gland, showed a remarkable improvement after rituximab treatment from baseline to week 48. We have not excluded the possibility that the IL-22 reduction is due to the natural progression of the disease, but our results indicated that rituximab therapy has the capacity to affect the immune-microenvironment of the inflamed glands of SS patients by decreasing IL-22 expression, which provides an additional immunological interpretation for rituximab efficacy in SS. Collectively, targeting IL-22 dysregulation may provide new insight into SS therapy.

### SSc

Systemic sclerosis (SSc) is a complex CTD characterized by fibrosis in any organ of the body, whose aetiology remains unknown. Abnormal interactions among fibroblasts, immunocytes and cells of the blood vessels may contribute to the pathogenesis of SSc. Fibroblasts are the cells that directly cause fibrosis. In SSc, the TGF-β pathways are activated, leading to the production of excessive amounts of collagens and proteoglycans. Then, extracellular matrix (ECM) is excessively deposited in tissues or organs, inducing fibrosis. A dysregulation of the fibrosis process may exist in the multi-systemic pathogenesis of SSc. Moreover, immune cells and several mediators may be involved in the complex process culminating in fibrosis. Notably, IL-22 produced by several immune cells is reported to contribute to SSc pathogenesis [72]. However, the exact mechanism initiating IL-22 activation is not clear. Thus, we reviewed current investigations of IL-22 in SSc, which may provide a potential therapeutic target for SSc patients.

#### The pathogenesis of IL-22 in SSc

The Mathian group found that circulating IL-22 levels were significantly increased in SSc patients relative to healthy controls [73]. Similarly, elevated IL-22 levels were observed in exhaled breath condensate and the serum of SSc patients [74]. Moreover, IL-22 transcripts were detected to be upregulated in skin samples from SSc patients compared with that in healthy controls, and there was a positive correlation between IL-22 transcription and extension of the skin involvement [73]. In addition, IL-22-producing cells were overexpressed in the epidermis of SSc patients. Interestingly, the Brembilla group demonstrated that the IL-22 receptor was expressed in SSc dermal fibroblasts [72]. Similarly, the Zhou Y group demonstrated that there was a positive correlation between the mRNA levels of the IL-22/IL-22R1 axis and the modified Rodnan skin score [75]. Most importantly, the Nicolò group showed that the elevated phosphorylation of MAP kinases and IL-22R downstream signalling were detected in dermal fibroblasts of SSc patients [72]. All of these studies indicated that IL-22 possibly played an important role in SSc pathogenesis.

Interestingly, the media from primary keratinocytes substantially prompted the production of MCP-1, MMP-1 and IL-8 by fibroblasts, suggesting that keratinocytes are able to release mediators that boost the process of fibrosis, as previously reported [76]. Furthermore, IL-22 has the capacity of prompting keratinocytes activated by TNF to induce fibroblast responses. The TNF/IL-22 axis and their receptors, together with TGF-β as a species of profibrotic cytokine, in part, constitute the existing inflammatory component of SSc [72]. These results indicate that keratinocyte–fibroblast interactions played a crucial role in the context of SSc [72]. In addition, increased expression of type I collagen protein was observed after stimulation with IL-22 in dermal fibroblasts of SSc patients, while there was no change in the IL-22 mRNA levels in dermal fibroblasts from healthy controls *in vitro* [77]. Consequently, IL-22 has the capacity to stimulate type I collagen expression post-transcriptionally, which differs from its normal transcriptional regulation (i.e., the overexpression of TGF-β/Smad mediated type I collagen). Furthermore, several microRNAs were indicated to be involved in regulating the expression of type I collagen protein by human dermal fibroblasts [78, 79]. Among them, let-7a negatively affected the expression of type I collagen [80]. Thus, the Soichiro group concluded that IL-22 induced type I collagen protein expression through downregulating let-7a, which contributed to the SSc pathogenesis [77] (Table 1).

As mentioned above, the proinflammatory action of IL-22 may trigger an indirect stimulus to the pathogenic process of fibrosis. However, a direct anti-fibrotic effect of IL-22 in SSc remains to be disclosed [81]. Additional research is necessary to uncover the real role of IL-22 in the pathogenesis of SSc.

#### Regulation of abnormal expression of IL-22 in SSc

Dysregulation of IL-22 contributes to SSc, and thus IL-22 could be a therapeutic target for this disorder. Marie-Elise et al. indicated that PGI_2_ analogues (i.e., iloprost, treprostinil and beraprost) markedly upregulated IL-22 expression in vitro [82]. PGI_2_ analogues decreased the regulation of lymphocyte adhesion to endothelial cells [83], and repressed the production of TNF-α by human monocytes [84], lymphocytes, and dendritic cells [85]. In the meantime, this investigation suggested that IL-22 acted as an anti-fibrotic mediator in SSc. In another study, J. Furuzawa et al. indicated that polymerized collagen has the capacity of downregulating the IL-22 level and other pro-inflammatory or profibrogenic cytokines (i.e., IL-17A and TGF-β1) in peripheral cells of SSc patients, renewing the skin architecture [86]. These investigations suggested that IL-22 may become a novel therapeutic avenue for SSc patients.

### DM

Dermatomyositis (DM) is an idiopathic inflammatory CTD whose primary feature is muscle weakness accompanied by increased skeletal muscle enzyme levels, characteristic electromyography and muscle biopsy findings. DM is divided into two types based on whether it involves the derma and myoglobin or not: DM and polymyositis (PM). Although the aetiology is not clear, activated Th1 and Th17 cells have been indicated to be involved in the pathophysiology of DM [87].

Cytokine IL-22, the effector of Th17 cells, may play a crucial role in the pathogenesis of DM. Francesco et al. found that a higher level of IL-22 protein was present in PM/DM patients and was positively correlated with myositis activity [88]. In the same study, IL-22R1 expression was observed in necrotic, degenerated myocytes and endothelial cells residing at the periphery of the inflammatory infiltrates, which implied that the IL-22/IL-22R axis may induce muscle damage [88]. The abnormal expression of the IL-22/IL-22R axis in endothelial cells may build a trans-endothelial bridge, which prompts the migration of inflammatory cells to the inflamed muscles. Moreover, the phosphorylated form of STAT3, due to IL-22R1 downstream signalling, was observable in L-22R1-expressing cells of DM patients. These results demonstrated that an autocrine inflammatory loop may exist in the involved muscles of DM patients [88]. Overall, IL-22/STAT3 signalling may offer therapeutic targets for DM (Table 1).

In view of a few studies about the pathogenesis of IL-22 in DM, additional studies are required to explore the precise mechanism of IL-22 in DM and to assess IL-22 whether expression can be a biomarker of treatment or prognosis for DM patients.

## IL-22, a double-edged sword

The dual nature of IL-22 is evident in the pathogenesis of several CTDs, such as RA and SSc. RA synovial fibroblasts (RASFs) can be prestimulated with IL-22, then co-cultured with human monocytes, which spurs those monocytes to differentiate into osteoclasts. These results showed that IL-22 has the potential to induce osteoclastogenesis by increasing the expression of nuclear factor κB ligand (RANKL) [89]. Moreover, the proliferation of cultured RASFs was increased and the expression of MCP-1 was upregulated after IL-22 treatment [44]. MCP-1, a pro-inflammatory mediator, recruits monocytes, dendritic cells and memory T cells to the site of inflammation, prolonging or amplifying the inflammatory process [90]. Nevertheless, in later stages of RA, IL-22 serum levels were observed to be negatively correlated with arthritis scores, and IL-22 administration locally decreased the inflammation level in the joints. These data indicated that IL-22 might be pathological in RA [91]. Notably, normal mice before the onset of RA showed a higher incidence and severity of arthritis after receiving anti-IL-22 treatment [49]. Moreover, higher scores of inflammations, synovitis, bone involvement, and cartilage destruction were detected in histopathologic examination of the paws. Taken together, IL-22 may play a dual role in RA: protective prior to the onset of RA and pathogenic after the onset of RA.

In addition, IL-22 may possess a dual nature in SSc [75]. It can be ‘pathological’ or ‘protective’ in SSc. For example, IL-22-deficient mice showed a significant reduction of lung inflammation and pulmonary fibrosis after high dose bleomycin treatment compared with IL-22-sufficient mice [92]. This result indicated IL-22 is pathological in SSc. Conversely, IL-22 produced by γδ T cells was shown to protect against lung fibrosis, using a mice model induced by hypersensitivity to *Bacillus subtilis* [81]. Thus, given the dual roles of IL-22 in CTDs, targeting different aspects of IL-22 may offer a novel therapeutic approach to CTDs.

## IL-22 as a potential therapeutic avenue for CTDs

In view of its pivotal roles in the cascade of inflammation and proliferation, IL-22 may have potential as a novel therapeutic for CTDs. It may be helpful for revealing therapeutic avenues to more fully comprehend the molecular mechanisms activating IL-22 and our current knowledge of immunosuppressive drugs to target this process. Promising results on experimental delivery of IL-22 have been reported for animal models of several CTDs. For example, to estimate the role of IL-22 in SLE, MRL/lpr mice, an SLE mouse model, were injected with anti-IL-22 mAb. The results suggested that anti-IL-22mAb administration to MRL/lpr mice that have not yet developed LN showed less proteinuria, less infiltration of inflammatory cells during the renal pathology and better renal function [31]. Moreover, in a SSc mouse model, a significant reduction of lung inflammation and pulmonary fibrosis was observed in the IL-22-deficient mice after they received high doses of bleomycin [92], indicating a pathogenic aspect of IL-22 in SSc. Similarly, to assess the role of IL-22 in RA, the Marijnissen group, using neutralizing anti-IL-22 antibodies, conducted a series of experiments in an RA mice model deficient in the IL-1 receptor antagonist (IL-1Ra^−/−^) [58]. Their results made it clear that the inflammation and bone erosion were significantly reduced in the IL-1Ra^−/−^mice after anti-IL-22 treatment. In addition, anti-cytokine vaccination is considered to be an innovative tactic for targeted active immunotherapy with potential practical applications in CTDs. All achievements mentioned above imply that IL-22 might be a potential therapeutic avenue for CTDs in the future.

## Conclusions

Accumulated data from both in vitro and in vivo models support the idea that IL-22 has therapeutic potential in CTDs. Development of recombinant IL-22, gene therapy delivery of IL-22, the discovery of antagonistic and agonistic antibodies of IL-22 or IL-22R, and the establishment of small chemical compounds that imitate IL-22 signaling for immunosuppression will be helpful to develop novel therapeutic approaches for CTDs. Notably, IL-22 acts as a double-edged sword in CTDs pathogenesis, which is the biggest barrier to developing therapeutic avenues based on this mediator. Consequently, additional studies are needed to explore the role of IL-22 in CTDs, especially in human systems. Moreover, the adverse events of any cytokine-associated therapy in CTDs should be illustrated in large studies.

## Data Availability

Not applicable.

## Abbreviations

RASF: RA synovial fibroblast
MCP-1: Monocyte chemoattractant protein 1
RANKL: Receptor activator of nuclear factor κB ligand
VIP: Vasoactive intestinal peptide
FLS: Fibroblast-like synoviocytes
TLR: Toll-like receptor
DM: Dermatomyositis
dcSSc: Diffuse cutaneous SSc
ECs: Epithelial cells
RF: Rheumatoid factor
HMGB1: Extracellular high mobility group box 1
SS: Sjögren’s syndrome
GCs: Glucocorticoids
CYC: Cyclophosphamide
HCQ: Hydroxychloroquine
LS: Lupus skin
LN: Lupus nephritis
TGF-β: Transforming growth factor-β
JAK1: Janus kinase-1
TYK2: Tyrosine kinase receptor-2
DCs: Dendritic cells
LPS: Lipopolysaccharide
CIA: Collagen-induced arthritis
NE: Norepinephrine
IRF4: Interferon regulatory factor 4
SSp: Primary SS
PM: Polymyositis
ICAM-1: Intercellular adhesion molecule 1
